# Identifying Problem-Talk and Co-rumination in Adolescents’ Social Networks

**DOI:** 10.1007/s10964-026-02428-4

**Published:** 2026-10-26

**Authors:** Sarah T. Malamut, Jack L. Andrews

**Affiliations:** 1 INVEST Flagship, University of Turku, Turku, Finland; 2 Department of Experimental Psychology, University of Oxford, Oxford, UK

**Keywords:** Co-rumination, Friendships, Internalizing symptoms, Friendship quality

## Abstract

Co-rumination (excessive problem-talk) is linked to adolescents’ psychosocial well-being. Traditional measures of co-rumination assess co-rumination tendencies with one (unspecified) friend. However, this approach overlooks both the number and characteristics of co-rumination partner(s). The current study identifies problem-talk partners and the degree to which individuals co-ruminate with each partner. The sample includes 579 Finnish adolescents (grades 4–9; M_age_ = 14.05, SD = 1.59; 52.3% self-identified as girl). More than half of adolescents (56%) nominated two or more problem-talk partners. Number of problem-talk partners and co-rumination among problem-talk partners were both associated with friendship closeness/satisfaction, but only co-rumination among problem-talk partners was positively linked to internalizing symptoms. Some evidence was found for a negative association between number of problem-talk partners and internalizing symptoms, underlining the importance of considering the specific ways in which problem-talk partners discuss problems.

## Introduction

Sharing one’s problems with others is a common experience, often forming the foundation of strong friendships. However, problem-talk may not always be beneficial, as it can also be characterized by excessive, repetitive discussion of problems and dwelling on negative feelings – a form of problem-talk known as co-rumination. Recent research has found a large degree of heterogeneity in the effects of co-rumination, as some adolescents experience benefits from co-rumination (e.g., increased friendship quality) while for others co-rumination can lead to higher internalizing symptoms (e.g., depression and anxiety) (DiGiovanni et al., 2021; Tilton‐Weaver & Rose, 2023). Importantly, there is evidence that adolescents’ co-rumination can lead to higher rates of internalizing problems not only for themselves, but also for their best friend (Schwartz-Mette & Rose, 2012). However, it is still unknown how co-rumination manifests across multiple friendships in adolescence, despite its important implications for adolescent well-being. Because co-rumination is a specific form of problem talk, the current study addresses this gap by first identifying adolescents’ problem-talk partners and assessing co-rumination within these relationships, and then examining how both number of problem-talk partners and extent of co-rumination with those partners are associated with the traditional co-rumination questionnaire, internalizing symptoms, and friendship satisfaction/closeness. This study also explores the degree of reciprocity among problem-talk partners, as well as whether reciprocated problem-talk relationships are characterized by greater dyadic co-rumination.

### Friendships, Problem-Talk, and Co-Rumination: Prior Findings and Limitations

As youth approach adolescence, friendships take on more importance and become an important source of social support, with self-disclosure and problem-talk becoming more frequent (Rose, 2007; Valkenburg et al., 2011). Consistent with the stress-buffering model, this social support derived from friendship is generally thought to mitigate the negative consequences of stressful events (Cohen & Wills, 1985; van Harmelen et al., 2017). Indeed, friends may provide adolescents with validation, advice, coping assistance, and reassurance that they are not alone - all of which may help with psychological well-being, even in the face of stressors. In addition, self-disclosure and seeking support from friends is known to foster intimacy between friends (Costello et al., 2024). Despite the potential benefits of self-disclosure and support seeking, recent research has found that friend support can sometimes actually intensify the consequences of certain stressors (e.g., Schacter et al., 2021).

One likely explanation for this surprising finding is that problem-talk with friends can be potentially maladaptive when these conversations involve excessive discussion of the problem and dwelling on negative feelings (i.e., co-rumination; Rose, 2002). Indeed, co-rumination is especially common among adolescents, who begin to rely more on friends for emotional support (Valkenburg et al., 2011) and for whom mental health problems are most likely to first emerge (Andrews et al., 2021; Solmi et al., 2022). Co-rumination is a form of problem-talk that may have developmental trade-offs (Rose, 2021). On the one hand, co-rumination is often linked to elevated friendship quality and intimacy, but on the other hand, it also involves dwelling on negative affect and is linked to internalizing symptoms. More recent research has painted an even more complicated picture, with some individuals solely benefiting from co-rumination, others experiencing only costs, while still others may experience both (e.g., DiGiovanni et al., 2021; Tilton‐Weaver & Rose, 2023).

Despite the important implications of problem-talk and co-rumination on adolescents’ well-being, there is still much that is not known regarding when friend support helps or hurts adolescents. This lack of knowledge may in part stem from how co-rumination is studied. The majority of existing studies of co-rumination focus only on the overall tendency for someone to co-ruminate with one (unidentified) friend. For example, most research on co-rumination has utilized self-reports of co-rumination (Rose, 2021) in which adolescents are required to answer questions with their same-sex best friend in mind. However, this approach does not reveal information about the friend with whom they co-ruminate, or whether both friends reciprocally share problems. In addition to these studies, other studies have utilized an observational approach which provides some information regarding the problem-talk partner. With this approach, two friends each discuss a personal problem together – demonstrating how the way in which friends discuss a problem during a conversation is associated with subsequent outcomes (e.g., mood and friendship closeness; Rose et al., 2014).

However, there are several key limitations to current approaches to studying problem-talk and co-rumination. Previous research on co-rumination has not taken into account that each adolescent may have more than one close, intimate friendship. A recent meta-analysis found that children and adolescents nominated an average of 5 friends, with variability shown when instructed to nominate “friends” (average = 7.34) vs. “best/close friends” (average = 3.76) (Neal, 2024). Thus, even if co-rumination only occurs in the closest or most intimate friendships, it is still likely that youth are engaging in this behavior or other forms of problem talk with more than one close friend. In support of this, a recent study found that adolescents reported 28.5% of their close friends also as friends with whom they spend a lot of time discussing problems (but did not assess co-rumination specifically; Schwartz et al., 2023).

### Problem-Talk and Co-Rumination Across Multiple Friendships

Rather than focusing only on adolescents’ problem-talk and co-ruminative tendencies within a single friendship, the implications of problem-talk and co-rumination may also depend on how many problem-talk partners adolescents have, the extent to which they co-ruminate with these problem-talk partners, and whether these relationships are reciprocated. Given that self-disclosure and problem-talk are features of friendships, it is likely that adolescents choose problem-talk partners out of their friends; however, many adolescents have several (close) friends, and much less is known regarding how many friends with whom adolescents discuss their problems or feelings. A greater number of problem-talk partners may signal having higher friendship quality, insofar as self-disclosure and problem-talk are more likely to occur among friendships of higher quality and closeness (e.g., Costello et al., 2024). Yet, more problem-talk partners may also reflect a tendency to be more emotion- or problem-focused (e.g., rumination) or greater emotional reliance on friends, both of which have been linked to internalizing problems (Nolen-Hoeksema et al., 2008; Rose et al., 2007). Alternatively, adolescents with a greater number of problem-talk partners may have more problems or stressors to discuss, and stressful life events in adolescence are associated with internalizing problems (March-Llanes et al., 2017).

Based on previous studies examining co-rumination (either via the traditional self-reported co-rumination questionnaire or observation studies), co-rumination with one friend has been linked with both friendship quality and internalizing symptoms. However, adolescents may co-ruminate with multiple friends and it is not yet clear whether similar patterns emerge when considering multiple problem-talk relationships. As co-rumination has not yet been assessed in this way, it is also important to examine whether this approach corresponds to the traditional co-rumination questionnaire, as well as the reliability of this approach over time.

Identifying the specific peers with whom adolescents discuss their problems also provides an opportunity to consider both the adolescents’ and partners’ perspectives. Indeed, there is currently little known regarding the extent to which perceived problem-talk relationships are reciprocated in adolescence, given that most previous studies have only included participants’ perspectives (or focused only on selected dyads). Consistent with developmental theories of friendship (e.g., Laursen & Hartup, 2002; Parker et al., 2006), reciprocity is a central feature of close friendships. Applied to problem-talk relationships, unreciprocated problem-talk nominations may reflect biased social-cognitive perceptions or mismatches in perceived intimacy or expectations of emotional support, which may be relevant for adolescents’ social and emotional well-being. Thus, reciprocity may be an important but understudied aspect of adolescents’ problem-talk relationships.

### Gender and Developmental Considerations

Both gender and age have emerged as two individual characteristics that play a key role in problem-talk and co-rumination (Rose, 2021). In general, girls’ friendships tend to be especially characterized by self-disclosure and problem-talk (Rose et al., 2016), and girls often report higher levels of co-rumination than boys (Rose, 2002). Moreover, girls tend to discuss more interpersonal problems with their friends compared to boys, which may be related to their higher levels of co-rumination, as co-rumination occurs more often when discussing interpersonal problems (Rose et al., 2022).

As friends become a more crucial source of support in adolescence compared to childhood, age may also play an important role in the dynamics of problem-talk and co-rumination. Previous studies have found that co-rumination is more prevalent in middle adolescence than late childhood (Rose, 2021). Moreover, in samples focused only on adolescence, co-rumination has still been positively associated with age, suggesting it may be more prevalent in middle adolescence even compared to early adolescence (e.g., Rose et al., 2017). However, there is mixed evidence whether specific dynamics of co-rumination (e.g., links with internalizing symptoms) actually differ as a function of adolescents’ age (e.g., early vs. mid-adolescence; Hankin et al., 2010).

## Current Study

Despite evidence that problem-talk involving co-rumination has important implications for adolescent well-being, existing research has largely examined co-rumination solely within one (often unspecified) friendship. As a result, less is known about how problem talk and co-rumination manifest across adolescents’ broader friendships. This study draws on peer nomination approaches to identify multiple problem-talk partners and, more specifically, measure the extent to which adolescents co-ruminate with each problem-talk partner. Number of problem-talk partners (Hypothesis 1) and mean dyadic co-rumination across problem-talk partners (Hypothesis 2) were each hypothesized to be positively associated with the traditional measure of co-rumination, internalizing symptoms, and friendship satisfaction/closeness. As an initial indicator of temporal reliability, both number of problem-talk partners and mean dyadic co-rumination were expected to be stable over time (Hypothesis 3). In addition to the main hypotheses, the current study also examined the reciprocity of problem-talk partner nominations as well as the extent to which problem-talk partner nominations corresponded with friendship nominations, as planned exploratory analyses. Given previous findings showing gender and developmental differences in co-rumination, the current study included planned sensitivity analyses controlling for gender and age. Additional exploratory analyses were also conducted to test for possible gender and age differences.

## Method

### Participants and Procedure

This data was collected as part of the DWELL project, with the purpose of identifying problem-talk partners and co-rumination in adolescents’ peer groups. Schools for this project were recruited by contacting the headmasters from a register of general educational institutions in Finland from 2024. To incentivize schools to participate, the schools were promised a report of their students’ well-being (completely anonymized) after the end of the study, as well as an iPad raffle for the students in their school. Using an online system that the schools use to communicate with parents/caregivers, information sheets and consent forms were sent to all eligible students (i.e., those in grades 4–9). Participants were Finnish youth in Grades 4–9 (*n* = 579; 52.3% self-identified as girl; 42.8% self-identified as boy; M_age_ = 14.05, SD = 1.59) across 6 schools (average of 7.5 participating students per class), who were present at school the day of data collection.

Participants completed online questionnaires during school in Spring 2025. In addition, a second wave of data was collected ~2–4 weeks later to replicate the findings at T1. Teachers supervised the administration of the questionnaires with detailed instructions. The survey software included variables that could be used to check unusual timing patterns. The students were assured that their answers were confidential, and informed that they could stop participating at any time. As students spend the majority of their time with the same classmates in the Finnish school system, classrooms were used as the reference group for all nomination-based variables (average classroom size = 18.17; see Measures below for more details), with all classmates (including non-participants) eligible for nomination (e.g., Marks et al., 2019). This procedure was in accordance with the ethical principles of Finnish National Board on Research Integrity (TENK) and the University of Turku Ethics Committee for Human Sciences. The hypotheses and analytic plan for main analyses were preregistered online at OSF (https://osf.io/n62tr/overview).

### Measures

#### Problem-talk Partners

Problem-talk partners can be defined as a network of peers within a classroom who discuss their problems with one another. At each wave, students were asked to nominate an unlimited number of (same- and cross-gender) classmates to the following question: “Which classmates do you discuss problems or your feelings with?” - all classmates were available to be nominated (except for self-nominations). This resulted in matrices for each classroom, with 0 = no nomination, 1 = nomination from one peer to another as a problem-talk partner. Number of problem-talk partners was calculated for each participant by summing the number of given problem-talk nominations.

#### Co-rumination

Two measures of co-rumination were included: mean dyadic co-rumination across problem-talk partners and the traditional co-rumination questionnaire. For each classmate that a participant nominated as a problem-talk partner, the participant also answered a series of questions about the classmate(s). Four items of co-rumination (responses ranging from 1 not at all true to 5 really true) were included: “When one of us has a problem, we talk about it for a long time”; “When one of us has a problem, we will keep talking about the problem even after we both know all of the details about what happened”; “When one of us has a problem, we talk a lot about all of the different bad things that might happen because of the problem”; and “When one of us has a problem, we talk for a long time about how upset it has made us.” For each nominated problem-talk partner, a dyadic co-rumination score was computed by averaging the four items. These scores were then averaged across nominated problem-talk partners to reflect each participant’s **mean dyadic co-rumination score** (αs = 0.88–0.92).

For the **traditional co-rumination questionnaire**, a short-form version (9 items; e.g., “We try to figure out every one of the bad things that might happen because of the problem”) of the self-report co-rumination questionnaire (CRQ; Rose, 2002) was used. Participants were provided the prompt: “These questions are about how you generally talk about your problems with your closest friend.” Responses ranged from 1 not at all true to 5 really true (see preregistration for full list of the 9 items used). A total score was computed by averaging the nine items (αs = 0.94–0.96).

#### Friendships

Participants were asked to choose an unlimited number of (same- and cross-gender) friends from their class roster, with the following question: “Which classmates are you friends with?” - all classmates were available to be nominated (except for self-nominations). This resulted in matrices for each classroom, with 0 = no nomination, 1 = nomination from one peer to another. For each classmate that a participant nominated as a friend, the participant also answered a series of questions about the classmate(s). **Friendship satisfaction** (“In general, how satisfied are you with your friendship?”; Morry, 2007), **perceived satisfaction of friend(s)** (“In general, how satisfied do you think your friend is with your friendship?”; Morry, 2007), and **friendship closeness** (“How close do you feel towards this friend?”; Lee, 2009) were assessed. For the two satisfaction items, responses ranged from 1 not satisfied to 7 very satisfied, and for friendship closeness 1 not very close to 7 extremely close. Each item was averaged across nominated friends, resulting in three indicators for each participant. The two satisfaction indicators were highly correlated (r = 0.80), and closeness was also strongly correlated with satisfaction (r = 0.65, p < 0.001). These indicators were therefore averaged to create a composite measure of friendship quality across friends. The three-item composite showed high internal consistency (αs = 0.85–0.91).

#### Internalizing Symptoms

Participants completed an 11-item measure (responses ranging from 0 = almost never to 3 = most of the time) assessing anxiety (e.g., “I worry when I go to bed at night”) and depressive symptoms (e.g., “I feel sad or empty”) (e.g., Garandeau et al., 2025; see preregistration for full list of 11 items). A total score for internalizing symptoms was computed by averaging the 11 items, with high internal consistency (αs = 0.92–0.94).

#### Control Variables

In sensitivity analyses, age and gender were added as control variables. Participants reported their self-identified gender. They were presented with four options: boy, girl, nonbinary and prefer not to answer. The majority of participants self-identified as girls (52.3%), followed by boys (42.8%), with 2.2% preferring not to answer, 1.0% self-identifying as non-binary, and 1.6% missing.

### Analytic Plan

All analyses were conducted in R (version 4.5.0) and statistical significance was evaluated at *p* < 0.05 for all pre-registered hypotheses. Across the main analyses, the unit of analysis was the adolescent, meaning each adolescent contributed one observation per model. To test Hypothesis 1, a series of linear mixed-effects models were run to examine whether the number of problem-talk partners (predictor) is associated with adolescents’ psychosocial outcomes. Separate models were estimated for each association of interest (traditional co-rumination questionnaire, internalizing symptoms and friendship satisfaction/closeness). Each model included random intercepts for classrooms and schools to account for non-independence of observations due to the hierarchical sampling structure. Sensitivity analyses were conducted controlling for student age and gender, with additional exploratory analyses testing for age and gender as possible moderators.

To test Hypothesis 2, additional linear mixed effects models were run, with mean dyadic co-rumination with problem-talk partners predicting each association of interest (traditional co-rumination questionnaire, internalizing symptoms, and friendship satisfaction/closeness). Sensitivity analyses were conducted controlling for student age and gender, with additional exploratory analyses testing for age and gender as possible moderators.

To test Hypothesis 3, Pearson’s r correlations were used to correlate scores between each time point. All categories for gender were retained for the initial sensitivity analyses with gender controlled for. However, the exploratory analyses including gender moderation were run on a slightly reduced sample, of adolescents who self-identified as a girl or boy, given the very small number of adolescents who reported being nonbinary or did not answer.

### Missing Data

Missingness on variables included in main analyses using Wave 1 was low, with less than 2% missing on most variables. The only exception was mean dyadic co-rumination, which could only be calculated for participants who nominated at least one problem-talk partner (*n* = 450) – as such the starting sample size was reduced for analyses with mean dyadic co-rumination as the predictor. Thus, missing data was handled by using all available cases for each model (i.e., when participants had valid data on all included variables). Model-specific analytic samples are provided in the Results tables.

### Power Analysis

Simulation-based sensitivity analyses were conducted to explore the standardized effect sizes that the multilevel models used in the current study were powered to detect. These analyses used the same multilevel structure as the main analyses, and evaluated standardized effect sizes ranging from β = 0.10 to β = 0.30. Power exceeded 0.80 for effects of approximately β = 0.15 or larger. Power to detect smaller effects of β = 0.10 was lower (0.56 to 0.76). Full simulation results can be found at the end of the Supplemental Material.

## Results

### Descriptive Statistics

Table 1 presents the participant demographics by school and Table 2 presents the means/SD and correlations between all study variables. Adolescents ranged from 0–13 given problem-talk partner nominations (22.2% with no nominated problem-talk partners, 21.8% with 1 nominated problem-talk partner, 20.2% with 2 nominated problem-talk partners and 36.8% with 3 or more nominated problem-talk partners).

**Table 1:** Descriptive statistics, depicting average age and gender of participants by school

School	School (N)	Classes (N)	Mean Age	SD Age	Boy	Girl	Nonbinary/Prefer not to say
Total	579	77	14.05	1.59	248	303	28
School 1	123	20	14.83	0.84	56	63	4
School 2	123	18	13.35	1.54	49	64	10
School 3	63	8	11.78	0.87	21	40	2
School 4	54	9	14.97	1.21	23	27	4
School 5	135	16	14.22	1.61	63	68	4
School 6	81	6	14.76	0.84	36	41	4

Only upper primary (grades 4–6), secondary (grades 7–9) or combined schools were eligible to participate. School 1 only had secondary students, School 3 only had primary students, and the other 4 schools were combined (grades 4–9)

**Table 2:** Means and SD, and correlations between study variables

	N	M	SD	1	2	3	4	5	6	7	8	9	10
**Variables at W1**													
1. Number of problem-talk partners	573	2.12	1.92	--	445	567	572	563	309	235	312	314	317
2. Mean dyadic co-rumination	446	3.49	0.97	−0.13^**^	--	443	444	444	243	217	243	246	249
3. Traditional co-rumination questionnaire	570	2.43	0.78	0.13^**^	0.62^***^	--	570	560	306	232	309	312	313
4. Internalizing symptoms	575	1.97	0.71	−0.12^**^	0.22^***^	0.30^***^	--	565	309	234	312	314	316
5. Friendship satisfaction/closeness	568	5.40	0.99	0.18^***^	0.20^***^	0.09^*^	−0.25^***^	--	306	234	309	311	315
**Variables at W2**													
6. Number of problem-talk partners	348	2.07	2.10	0.62^***^	0.06	0.19^**^	−0.10	0.21^***^	--	259	339	342	334
7. Mean dyadic co-rumination	259	3.46	0.96	0.01	0.60^***^	0.54^***^	0.17^*^	0.12	0.02	--	252	254	257
8. Traditional co-rumination questionnaire	352	2.37	0.88	0.09	0.43^***^	0.64^***^	0.22^***^	0.07	0.16^**^	0.65^***^	--	349	339
9. Internalizing symptoms	354	1.94	0.77	−0.09	0.18^**^	0.28^***^	0.79^***^	−0.17^**^	−0.06	0.22^***^	0.32^***^	--	341
10. Friendship satisfaction/closeness	354	5.49	1.09	0.20^***^	0.14^*^	0.08	−0.13^*^	0.57^***^	0.17^**^	0.30^***^	0.16^**^	−0.13^*^	--

Below the diagonal are Pearson correlations of the variables at W1 and W2; above the diagonal the pairwise sample sizes are presented. Pairwise sample sizes for correlations ranged from n = 217 to n = 572. ^*^p < 0.05, ^**^p < 0.01, ^***^p < 0.001

**Hypothesis 1: Associations with the number of problem-talk partners**

To test the first hypothesis, the association between number of problem-talk partners and the traditional co-rumination questionnaire, internalizing symptoms, and friendship satisfaction/closeness was examined (Table 3). Number of problem-talk partners was positively and significantly associated with the traditional co-rumination questionnaire and friendship satisfaction/closeness. Number of problem-talk partners, however, was significantly negatively associated with internalizing symptoms. The pattern of findings remained consistent when controlling for age and gender (see Table S1) and were replicated at Wave 2 (see Table S3), with one exception. At Wave 2, the negative association between number of problem-talk partners and internalizing symptoms was only significant when also controlling for age and gender.

**Table 3:** Linear mixed-effects models for Hypothesis 1. The number of problem-talk partners nominated in each class predicting traditional co-rumination questionnaire, internalizing symptoms and friendship satisfaction/closeness

	Traditional co-rumination questionnaire					Internalizing symptoms					Friendship satisfaction/closeness				
*Predictors*	*b*	*SE*	95% CI	${\rm{\beta }}$	*p*	*b*	*SE*	95% CI	${\rm{\beta }}$	*p*	*b*	*SE*	95% CI	${\rm{\beta }}$	*p*
(Intercept)	2.33	0.05	[2.22, 2.43]		<**0.001**	2.06	0.04	[1.98, 2.15]		<**0.001**	5.19	0.06	[5.07, 5.32]		<**0.001**
Problem-talk partners	0.05	0.02	[0.02, 0.09]	0.13	**0.002**	−0.05	0.02	[−0.08, −0.02]	−0.12	**0.003**	0.10	0.02	[0.05, 0.14]	0.19	<**0.001**
**Random Effects**															
σ^2^	0.55					0.49					0.92				
τ_00_	0.03_class_					0.00_class_					0.03_class_				
	0.00_school_					0.00_school_					0.00_school_				
ICC	--					--					--				
N	6_school_					6_school_					6_school_				
	77_class_					77_class_					77_class_				
Observations	567					572					563				
Marginal R^2^ / Conditional R^2^	0.017 / NA					0.016 / NA					0.035 / NA				

*b*, *SE*, 95% CIs, and *p* values are from unstandardized mixed-effects models. β values are standardized estimates and are presented only for continuous predictors. Random-effect variance components are rounded to two decimals

**Hypothesis 2: Co-rumination with problem-talk partners**

To test the second hypothesis, the extent to which adolescents co-ruminated with their problem-talk partners was associated with the traditional co-rumination questionnaire, internalizing symptoms, and friendship satisfaction/closeness was examined. Participants were included in this analysis if they nominated at least one problem-talk partner. Adolescents who reported co-ruminating more intensely with their problem-talk partners scored higher on the traditional co-rumination questionnaire, internalizing symptoms, and friendship satisfaction/closeness (see Table 4). All results held when controlling for age and gender (see Table S2) and were replicated at Wave 2 (see Table S3).

**Table 4:** Linear mixed-effects models for Hypothesis 2. The mean dyadic co-rumination score (i.e., the average score among each individual reporting the level of co-rumination they engage in with their problem-talk partners) predicting traditional co-rumination questionnaire, internalizing symptoms and friendship satisfaction/closeness

	Traditional co-rumination questionnaire					Internalizing symptoms					Friendship satisfaction/closeness				
*Predictors*	*b*	*SE*	95% CI	${\rm{\beta }}$	*p*	*b*	*SE*	95% CI	${\rm{\beta }}$	*p*	*b*	*SE*	95% CI	${\rm{\beta }}$	*p*
(Intercept)	0.85	0.11	[0.64, 1.06]		<**0.001**	1.40	0.12	[1.15, 1.64]		<**0.001**	4.78	0.17	[4.45, 5.10]		<**0.001**
Mean dyadic co-rumination	0.49	0.03	[0.43, 0.55]	0.62	<**0.001**	0.16	0.03	[0.09, 0.23]	0.22	<**0.001**	0.20	0.05	[0.11, 0.29]	0.20	<**0.001**
**Random Effects**															
σ^2^	0.34					0.47					0.85				
τ_00_	0.01_class_					0.00_class_					0.01_class_				
	0.00_school_					0.00_school_					0.00_school_				
ICC	--					0.00					--				
N	6_school_					6_school_					6_school_				
	76_class_					76_class_					76_class_				
Observations	443					444					444				
Marginal R^2^ / Conditional R^2^	0.387 / NA					0.048 / 0.052					0.041 / NA				

*b*, *SE*, 95% CIs, and *p* values are from unstandardized mixed-effects models. β values are standardized estimates and are presented only for continuous predictors. Random-effect variance components are rounded to two decimals

**Hypothesis 3: Stability across time**

To test the third hypothesis, the extent to which number of problem-talk partners and degree of co-rumination with problem-talk partners remained stable across both waves of data collection was examined. Among participants with valid problem-talk data at both waves (*n* = 309), the number of problem-talk partners was positively correlated across waves (*r* = 0.62, *p* < 0.001). On average, participants retained 75.3% of their Wave 1 problem-talk partners at Wave 2, and 81.7% of Wave 2 problem-talk partners had also been nominated by the same participant at Wave 1. In addition, of participants who nominated at least 1 problem-talk partner at both waves, 98.6% nominated at least one of the same partners across waves. Mean dyadic co-rumination with nominated problem-talk partners was also positively correlated across waves (*r* = 0.60, *p* < 0.001), among participants with valid dyadic co-rumination scores at both waves (*n* = 217). Together, these findings support the short-term stability of both the number of problem-talk partners adolescents nominated and the extent to which adolescents reported co-ruminating with those partners.

### Preregistered Exploratory Analyses

#### Reciprocation of Problem-Talk Partners

As planned exploratory analyses, the reciprocity of problem-talk nominations was explored. Reciprocity analyses were limited to ties in which both adolescents were participants in the study, as reciprocity could not be accurately evaluated for nominations to non-participating classmates. Among 690 problem-talk nominations between participating students, 434 of these were reciprocated (62.9%) with 256 being unilateral nominations. Still focused only on ties between participants, adolescents reported higher dyadic co-rumination with reciprocated problem-talk partners (mean dyadic co-rumination: 3.61, *SD* = 0.99) than when their problem-talk tie was not reciprocated (mean dyadic co-rumination: 3.09, *SD* = 1.04), *t*(511) = −6.49, *p* < 0.001, *d* = 0.51. Because these directed ties were not independent, this difference was also explored using a tie-level mixed model with random intercepts for nominators and nominees; reciprocated ties had significantly higher dyadic co-rumination than one-directional ties, *b* = 0.41, *SE* = 0.06, *p* < 0.001.

Given that mean dyadic co-rumination was higher in reciprocated ties, additional person-level sensitivity analyses were conducted to explore whether the findings with mean dyadic co-rumination as the predictor (H2) were driven by adolescents with reciprocated problem-talk nominations. Mean dyadic co-rumination remained significantly associated with the traditional co-rumination questionnaire, internalizing symptoms, and friendship satisfaction/closeness, after controlling for whether adolescents had at least one reciprocated problem-talk partner.

#### Correspondence between Problem-talk Partners and Friendship Nominations

Of participants’ nominated problem-talk partners, 98.4% were also nominated as friends. Adolescents reported problem talking with approximately 40% (39.95%) of their nominated friends. 22% of adolescents reported not problem talking with any of their nominated friends.

### Additional Exploratory Analyses

In addition to the above preregistered exploratory analyses, a series of additional exploratory analyses were conducted to check the robustness of the findings. Outputs from these analyses are available in the Supplemental Material.

#### Gender and Age Moderation

Given prior evidence that problem-talk and co-rumination may vary by gender and age, exploratory moderation analyses were conducted. All continuous variables included in the interaction terms were grand-mean centered before calculating the interaction terms. For these models, the sample was limited to those who self-identified as a boy or girl (with boys as the reference group), given the small number of students who gave a different answer. First, gender and age were tested as moderators of the association between number of problem-talk partners and each outcome (the traditional co-rumination questionnaire, internalizing symptoms, and friendship satisfaction/closeness). There was no evidence that the associations between number of problem-talk partners and the outcomes significantly differed by gender or age. This was then repeated with mean dyadic co-rumination as the predictor instead of number of problem-talk partners. Likewise, there was no evidence that the link between mean dyadic co-rumination and the outcomes differed by gender or age. Estimates, standard errors, 95% CI, *p* values and model-specific sample sizes can be found for each interaction term in Table S4.

#### Number of Friends

An alternative explanation for Hypothesis 1 is that the number of problem-talk partners may simply reflect having more friends. To address this possibility, number of friends was added as an additional control variable to the Hypothesis 1 models shown in Table S1. When controlling for number of friends, the pattern of findings for Hypothesis 1 remained unchanged: number of problem-talk partners remained positively associated with the traditional co-rumination questionnaire and friendship satisfaction/closeness, and negatively associated with internalizing symptoms (Table S5).

#### CR2 Robust Standard Errors

Although the primary analyses included school and classroom random intercepts, these random intercepts do not fully address the possibility of residual dependence among adolescents within a classroom (e.g., due to being friends or problem-talk partners). Thus, additional analyses were conducted using linear models with school-level fixed effects and CR2 robust standard errors clustered by classroom. The pattern of findings in these models remained unchanged (Table S6).

#### Classroom Participation Rates

Lastly, additional analyses were conducted to check that the findings were robust to variations in classroom participation rates. Classroom participation rate did not significantly moderate any of the associations tested in Hypotheses 1 and 2. None of these interactions were significant, suggesting the primary associations did not differ as a function of classroom participation rate (Table S7). In addition, because the lower end of the classroom participation range was driven by one outlier classroom, the main analyses were rerun with this classroom excluded, with the pattern of findings remaining unchanged.

## Discussion

Friendships become increasingly important and intimate in adolescence, with higher prevalence of self-disclosure and seeking support from close friends, relative to childhood (e.g., Buhrmester & Prager, 1995; Parker et al., 2006). Although social support from friends is often thought to protect against the negative consequences of stressors, friends can also excessively dwell on problems and negative feelings with one another: a form of problem-talk known as co-rumination. This can be problematic, as co-rumination can sometimes inadvertently exacerbate mental health problems (van Harmelen et al., 2017). Despite the important implications of problem-talk and co-rumination on adolescents’ well-being, previous research has not accounted for the likelihood that adolescents may have multiple close friends with whom they may problem-talk or co-ruminate, or examined the degree to which problem-talk relationships are reciprocated in adolescence. The current study addressed these gaps by identifying which peers adolescents nominated as problem-talk partners, as well as the extent to which they co-ruminated with those partner(s). This study examined whether the number of problem-talk partners and co-rumination with these partner(s) was linked to the traditional co-rumination questionnaire, internalizing symptoms and friendship satisfaction/closeness, with all analyses replicated across two time points. In addition, the current study demonstrated that more than half of adolescents reported problem-talk with more than one friend. Future research on problem-talk and co-rumination should consider these processes across multiple friendships in adolescence, especially given that number of problem-talk partners and co-rumination among problem-talk partners were differentially associated with internalizing problems.

As predicted, adolescents who had more problem-talk partners also reported higher levels on the traditional co-rumination questionnaire and self-reported higher levels of friendship satisfaction/closeness. These findings are consistent with past research highlighting self-disclosure and support seeking as markers of high-quality friendships (e.g., Costello et al., 2025). Contrary to expectations, the number of problem-talk partners was negatively associated with internalizing symptoms in the main analyses. However, the extent to which adolescents co-ruminated with their problem-talk partners was positively associated with internalizing symptoms. This supports the idea that engaging in problem-talk itself (even with multiple friends) may not inherently be linked to risks to one’s mental health (i.e., elevated internalizing symptoms), but rather that the specific ways in which adolescents discuss their problems are related to one’s mental health. Taken together, this suggests that engaging in problem-talk itself may actually be a protective factor. One possible alternative explanation is that this finding could also reflect benefits of adolescents having a greater number of friendships that are intimate enough to include problem-talk. However, to explore this, additional models were run controlling for number of friends, with the negative association between number of problem-talk partners and internalizing symptoms remaining. In addition, given that prevalence of problem-talk and co-rumination can vary considerably by gender or age (Rose, 2021), possible gender and age moderation was also explored; however, no support was found for gender or age differences.

### Problem-Talk Partners and Reciprocity

In exploratory analyses, the current study found that adolescents nominated over a third (40%) of their friends as problem-talk partners. This shows that even though adolescents may problem-talk with multiple friends, they may still selectively choose among their friends when deciding who to entrust with their problems. For example, adolescents may only go to their closest friends or those with whom they feel most secure when they want to discuss their problems. By asking adolescents to separately nominate their friends and those with whom they problem-talk, this approach opens avenues for future research to examine which specific friends adolescents are more or less likely to problem-talk with, as well as tie-level characteristics of problem-talk partners (e.g., same- vs. cross-gender friends).

When considering possible reciprocal nominations (i.e., between two participating students), over a third (37%) of adolescents’ problem-talk partner nominations were *not* reciprocated. Reciprocated problem-talk dyads tended to also engage in higher levels of co-rumination with one another, compared to unreciprocated problem-talk ties. This is an important finding, as most research on co-rumination focuses solely on the participant’s perspective of the extent to which they (and the friend they have in mind) engage in various co-ruminative behaviors – however, the friend may have a different perception of their encounters. There are several reasons for these findings. For example, some adolescents may not be very good at identifying with whom they problem-talk, or it might be that if the problem talk is entirely one-sided (i.e., driven by one adolescent), the other friend may not identify the adolescent as someone they seek out for support. Relatedly, this finding may also reflect adolescents who engage in high levels of conversational self-focus (i.e., the tendency to redirect conversations about problems to focus on the self; Schwartz-Mette & Rose, 2016).

In addition, although participants’ own perceptions of their relationships and interaction patterns are highly relevant for understanding links between co-rumination and mental health, relying solely on self-reports may not fully capture the extent to which problem-talk (and co-rumination more specifically) occurs or relates to well-being. In particular, adolescents with pre-existing internalizing problems may be more likely to self-report elevated levels of co-rumination (e.g., due to shared method bias), independent of the actual frequency or intensity of problem-focused discussions with their friend(s). This may contribute to the finding of some discrepancies between adolescents’ reports and their friends’ perspectives on whether they are problem-talk partners. Future research could build on this work by examining whether adolescents and their problem-talk partner(s) agree not only that their relationship involves problem-talk, but also whether they agree about the problem-talk strategies they tend to use together (such as co-rumination). With the approach used in this study (i.e., identifying both the nominators’ and nominees’ perspectives), future research could also examine the consequences (e.g., friendship quality, friendship stability) of (mis)matched perceptions among friends regarding whether they are problem-talk partners or their tendencies towards specific strategies.

### Limitations & Future Research

Whilst these results demonstrate the added benefit of the peer nomination approach to measuring co-rumination among adolescents, it should be acknowledged that peer nomination data can be more burdensome to collect, which may partially be why studies often rely on self-reported co-rumination. Indeed, school-based research focused on sociometric data faces many challenges; however, there are ways to overcome these challenges (e.g., offering to share (anonymized) findings with schools; compromising with schools regarding included items: see Mayeux & Kraft, 2017). Despite potential challenges, this type of data is necessary to better understand problem-talk dynamics across adolescents’ friendships. Another concern related to online school-based data collection is the possibility of careless responding (e.g., Curran, 2016). Although surveys were completed online, data collection occurred in a structured school setting rather than at home unsupervised. The data also included items to check unusual timing data in responses. However, as the survey did not include instructed-response or attention-check items, careless responding cannot be ruled out entirely.

In addition, a common limitation of school-based data collections is low participation rate in classrooms, which can negatively affect the validity and reliability of peer nomination items (Cillessen & Marks, 2011) – especially when examining received nominations or peer reputation in the classroom. Although some classrooms in this sample had low participation rates, this concern is less directly applicable to the primary analyses of the current study, which focused on adolescents’ given nominations and partner-specific ratings using the full classroom roster of all classmates. Sensitivity analyses indicated that the key results did not depend on classroom participation rate and also remained consistent when excluding an outlier classroom. However, low classroom participation rate is especially relevant for the exploratory reciprocation analyses, as reciprocity could only be observed when both members of a potential problem-talk relationship participated. As such, these exploratory analyses had to rely on a reduced sample.

The novel approach to examining co-rumination, which captures multiple possible problem-talk ties within a peer group, also provides an opportunity to discover if and how co-rumination spreads in a peer group. For example, future research with larger samples with several waves could select classrooms meeting certain criteria (i.e., high enough participation rate) and use social network analysis for a range of research questions, including whether adolescents with a shared problem-talk partner subsequently begin to co-ruminate together. Given that mental health problems can be transmitted within peer networks (e.g., Alho et al., 2024), this approach could be a valuable tool in investigating possible mechanisms by which this process occurs.

Future work could also build on this study by examining the extent to which adolescents use various problem-talk strategies with their friends, some of which may be positive. In the current study, only co-rumination among problem-talk partners was positively associated with internalizing symptoms, but not the number of problem-talk partners; in fact, number of problem-talk partners was negatively associated with internalizing symptoms. This suggests that it is important to distinguish between having problem-talk partners, and the extent to which problem-talk partners engage in different strategies (e.g., co-problem-solving, co-distracting). It is possible that problem-talk partners use several strategies (as can be observed in individuals; e.g., Dixon-Gordon et al., 2015), or it is also possible that adolescents go to some friends for a specific strategy (e.g., when they want to vent or co-ruminate) and another friend for more positive, or solution focused strategies (e.g., when they want advice or to problem-solve).

Moreover, future research could investigate whether the use of other – perhaps more adaptive – strategies offset potential consequences of co-rumination, or whether co-rumination is still associated with internalizing symptoms even when friends use other strategies as well. Given the cross-sectional nature of the current study, future research should also examine prospective associations between adolescents’ use of different strategies with specific problem-talk partners and subsequent psychosocial well-being. In addition, the current study focused on internalizing symptoms more broadly rather than clinical levels. Worry, sadness, and other related emotional experiences are common during adolescence and may reflect normative responses to different life stressors. Future research can further examine not only how different strategies with specific problem-talk partners are correlated with emotional symptoms, but also if and when these strategies may elevate or reinforce (normative) emotional responses in a way that may put adolescents at risk for more severe difficulties.

In the current study, the included items to assess co-rumination between problem-talk partners focused on how the pair typically behaves when one of them has a problem, as the focus was on general tendencies within different friendships. However, assessing this at the dyadic level (e.g., “*we* talk about it for a long time”) does not clarify individual contributions to the ruminative nature of the conversation (DiGiovanni et al., 2026). For example, one individual may seek out social rumination with their friend, but that does not necessarily mean that the behaviors are reciprocated by that friend (e.g., social rumination vs. co-rumination; DiGiovanni et al., 2026). Future research could build on this approach by differentiating between what the individual tends to do (and/or what they perceive their partner to do) for each problem-talk partner. Furthermore, although the current study primarily focused on the participants’ report of dyadic co-rumination ratings for each partner, future research could further consider the (mis)match between nominators’ and nominees’ perceptions. Whereas the current study examined agreement about whether a problem-talk relationship exists, future research could build on this by assessing whether problem-talk partners agree on the extent to which they use specific problem-talk strategies (e.g., co-rumination) within their relationship.

Lastly, the current study focused on a sample of Finnish adolescents, with a focus on classmates. Although there are not clear reasons to suspect the findings would be specific to a Finnish sample, future research is needed in other cultures and contexts as well, to ensure that the findings are generalizable. Future research could also consider identifying problem-talk partners with a different reference group, such as all grademates.

## Conclusion

Research on co-rumination has primarily focused on adolescents’ tendency to co-ruminate with one, unspecified (often same-sex) best friend. However, this overlooks the extent to which adolescents problem-talk and co-ruminate with multiple friends, as well as the degree to which problem-talk relationships are reciprocated in adolescence. To address this gap, the current study assessed which classmates adolescents report as problem-talk partners, as well as the degree to which they co-ruminate with these partners. Using this approach, the current study found that more than half of adolescents nominated two or more problem-talk partners. Moreover, adolescents do not consider all of their friends as also being problem-talk partners, and over a third of problem-talk partner nominations were not reciprocated. Number of problem-talk partners, as well as the extent to which they co-ruminate with their problem-talk partner(s) was positively associated with the traditional co-rumination questionnnaire and friendship closeness/satisfaction. Only co-rumination among problem-talk partners, but not number of problem-talk partners, was positively associated with internalizing problems. However, evidence was found for a negative association between number of problem-talk partners and internalizing symptoms, underlining the importance of considering the specific ways in which problem-talk partners discuss problems. The approach used in the current study opens a new avenue for research questions, including how problem-talk and co-rumination are embedded within adolescents’ broader peer networks.

## Supplementary information

Supplemental Material.R2
