# Supplementary material for: Identifying Problem-Talk and Co-rumination in Adolescents’ Social Networks
Source: J Youth Adolesc. 2026 Oct 26;55(11):2705–17. doi: 10.1007/s10964-026-02428-4 (PMC13768393; doi:10.1007/s10964-026-02428-4)
Supplement: Supplementary file 1 [file 10964_2026_2428_MOESM1_ESM.docx]

**Identifying Problem-Talk and Co-Rumination in Adolescents’ Social Networks**

**Supplemental Material**

Table S1. Sensitivity analysis for each model run for Hypothesis 1, controlling for gender and age.

Table S2. Sensitivity analysis for each model run for Hypothesis 2, controlling for gender and age.

Table S3. Replication of base and gender/age-adjusted models for Hypothesis 1 and 2, on Wave 2 data.

Table S4. Gender and age moderation of Hypothesis 1 and 2 associations.

Table S5. Number of friends as an additional control variable for Hypothesis 1 associations.
Table S6. CR2 robust standard error sensitivity analyses for Hypothesis 1 and 2.

Table S7. Classroom participation rate as a moderator of Hypothesis 1 and 2 associations.

Table S8. Simulation-based power for Hypothesis 1 and 2.

Table S1. Sensitivity analysis for each model run for Hypothesis 1, controlling for gender and age.

|  | **Traditional co-rumination questionnaire** | | | | | **Internalizing symptoms** | | | | | **Friendship satisfaction/closeness** | | | | |
| --- | --- | --- | --- | --- | --- | --- | --- | --- | --- | --- | --- | --- | --- | --- | --- |
| *Predictors* | *b* | *SE* | 95% CI | $\beta$ | *p* | *b* | *SE* | 95% CI | $\beta$ | *p* | *b* | *SE* | 95% CI | $\beta$ | *p* |
| (Intercept) | 2.10 | 0.06 | [1.98, 2.22] |  | **<.001** | 1.76 | 0.05 | [1.66, 1.86] |  | **<.001** | 5.22 | 0.08 | [5.06, 5.38] |  | **<.001** |
| Problem-talk partners | 0.05 | 0.02 | [0.02, 0.08] | 0.13 | **.003** | -0.05 | 0.01 | [-0.08, -0.02] | -0.13 | **.001** | 0.10 | 0.02 | [0.05, 0.14] | 0.18 | **<.001** |
| Gender (girl) | 0.43 | 0.06 | [0.30, 0.55] |  | **<.001** | 0.53 | 0.06 | [0.42, 0.64] |  | **<.001** | -0.04 | 0.09 | [-0.21, 0.12] |  | .619 |
| Gender (NB) | 0.51 | 0.34 | [-0.15, 1.17] |  | **.130** | 1.42 | 0.29 | [0.85, 2.00] |  | **<.001** | -0.65 | 0.49 | [-1.62, 0.32] |  | .189 |
| Gender (prefer not to answer) | -0.13 | 0.25 | [-0.62, 0.36] |  | **.605** | 0.66 | 0.20 | [0.27, 1.05] |  | **<.001** | 0.07 | 0.30 | [-0.52, 0.65] |  | .824 |
| Age | 0.05 | 0.02 | [0.00, 0.10] | 0.10 | **.035** | 0.02 | 0.02 | [-0.01, 0.06] | 0.05 | .228 | -0.03 | 0.03 | [-0.08, 0.03] | -0.04 | .375 |
| **Random Effects** | | | | | | | | | | | | | | | |
| σ^2^ | 0.51 | | | | | 0.41 | | | | | 0.92 | | | | |
| τ_00_ | 0.03 _class_ | | | | | 0.00 _class_ | | | | | 0.02 _class_ | | | | |
|  | 0.00 _school_ | | | | | 0.00 _school_ | | | | | 0.00 _school_ | | | | |
| ICC | -- | | | | | -- | | | | | -- | | | | |
| N | 6 _school_ | | | | | 6 _school_ | | | | | 6 _school_ | | | | |
|  | 77 _class_ | | | | | 77 _class_ | | | | | 77 _class_ | | | | |
| Observations | 552 | | | | | 557 | | | | | 549 | | | | |
| Marginal R^2^ / Conditional R^2^ | 0.100 / NA | | | | | 0.174 / NA | | | | | 0.041 / NA | | | | |

*Note. b*, *SE*, 95% CIs, and *p* values are from unstandardized mixed-effects models. β values are standardized estimates and are presented only for continuous predictors. Gender was entered as a four-level factor with boys as the reference group (NB = nonbinary). Random-effect variance components are rounded to two decimals.

Table S2. Sensitivity analysis for each model run for Hypothesis 2, controlling for gender and age.

|  | **Traditional co-rumination questionnaire** | | | | | **Internalizing symptoms** | | | | | **Friendship satisfaction/closeness** | | | | |
| --- | --- | --- | --- | --- | --- | --- | --- | --- | --- | --- | --- | --- | --- | --- | --- |
| *Predictors* | *b* | *SE* | 95% CI | $\beta$ | *p* | *b* | *SE* | 95% CI | $\beta$ | *p* | *b* | *SE* | 95% CI | $\beta$ | *p* |
| (Intercept) | 0.84 | 0.11 | [0.63, 1.05] |  | **<.001** | 1.28 | 0.12 | [1.05, 1.52] |  | **<.001** | 4.82 | 0.17 | [4.48, 5.15] |  | **<.001** |
| Mean dyadic co-rumination | 0.48 | 0.03 | [0.42, 0.53] | 0.60 | **<.001** | 0.11 | 0.03 | [0.05, 0.18] | 0.15 | **<.001** | 0.22 | 0.05 | [0.13, 0.31] | 0.22 | **<.001** |
| Gender (girl) | 0.10 | 0.06 | [-0.01, 0.22] |  | **.080** | 0.46 | 0.07 | [0.33, 0.59] |  | **<.001** | -0.17 | 0.09 | [-0.36, 0.01] |  | .066 |
| Gender (NB) | 1.34 | 0.59 | [0.18, 2.49] |  | **.023** | 1.65 | 0.65 | [0.37, 2.93] |  | **.012** | -0.16 | 0.92 | [-1.98, 1.65] |  | .861 |
| Gender (prefer not to answer) | -0.33 | 0.21 | [-0.74, 0.09] |  | .124 | 0.70 | 0.24 | [0.24, 1.16] |  | **.003** | -0.50 | 0.33 | [-1.15, 0.15] |  | .134 |
| Age | 0.03 | 0.02 | [-0.01, 0.06] | 0.05 | .176 | 0.03 | 0.02 | [-0.01, 0.07] | 0.06 | .206 | -0.04 | 0.03 | [-0.10, 0.01] | -0.07 | .136 |
| **Random Effects** | | | | | | | | | | | | | | | |
| σ^2^ | 0.34 | | | | | 0.42 | | | | | 0.84 | | | | |
| τ_00_ | 0.00 _class_ | | | | | 0.00 _class_ | | | | | 0.01 _class_ | | | | |
|  | 0.00 _school_ | | | | | 0.00 _school_ | | | | | 0.00 _school_ | | | | |
| ICC | -- | | | | | -- | | | | | -- | | | | |
| N | 6 _school_ | | | | | 6 _school_ | | | | | 6 _school_ | | | | |
|  | 76 _class_ | | | | | 76 _class_ | | | | | 76 _class_ | | | | |
| Observations | 436 | | | | | 437 | | | | | 437 | | | | |
| Marginal R^2^ / Conditional R^2^ | 0.405 / NA | | | | | 0.160 / NA | | | | | 0.055 / NA | | | | |

*Note. b*, *SE*, 95% CIs, and *p* values are from unstandardized mixed-effects models. β values are standardized estimates and are presented only for continuous predictors. Gender was entered as a four-level factor with boys as the reference group (NB = nonbinary). Random-effect variance components are rounded to two decimals.

Table S3. Replication of base and gender/age-adjusted models for Hypothesis 1 and 2, on Wave 2 data.

| **Hypothesis** | **Outcome** | **Predictor** | **Model** | ***b*** | **95% CI** | ***p*** | **Consistent with Wave 1** |
| --- | --- | --- | --- | --- | --- | --- | --- |
| H1 | Traditional co-rumination | Problem-talk partners | Base | 0.06 | [0.02, 0.11] | <.001 | Yes |
| H1 | Traditional co-rumination | Problem-talk partners | Gender/age-adjusted | 0.07 | [0.03, 0.11] | .002 | Yes |
| H1 | Internalizing symptoms | Problem-talk partners | Base | -0.02 | [-0.06, 0.01] | 0.21 | No |
| H1 | Internalizing symptoms | Problem-talk partners | Gender/age-adjusted | -0.05 | [-0.08, -0.01] | .018 | Yes |
| H1 | Friendship satisfaction/closeness | Problem-talk partners | Base | 0.09 | [0.04, 0.15] | <.001 | Yes |
| H1 | Friendship satisfaction/closeness | Problem-talk partners | Gender/age-adjusted | 0.11 | [0.05, 0.16] | <.001 | Yes |
| H2 | Traditional co-rumination | Mean dyadic co-rumination | Base | 0.56 | [0.49, 0.64] | <.001 | Yes |
| H2 | Traditional co-rumination | Mean dyadic co-rumination | Gender/age-adjusted | 0.54 | [0.46, 0.63] | <.001 | Yes |
| H2 | Internalizing symptoms | Mean dyadic co-rumination | Base | 0.17 | [0.08, 0.26] | <.001 | Yes |
| H2 | Internalizing symptoms | Mean dyadic co-rumination | Gender/age-adjusted | 0.14 | [0.05, 0.24] | .003 | Yes |
| H2 | Friendship satisfaction/closeness | Mean dyadic co-rumination | Base | 0.31 | [0.19, 0.44] | <.001 | Yes |
| H2 | Friendship satisfaction/closeness | Mean dyadic co-rumination | Gender/age-adjusted | 0.32 | [0.19, 0.45] | <.001 | Yes |

*Note.* The pattern of findings was consistent with Wave 1 for all models, with the exception of the base model testing the link between number of problem-talk partners and internalizing symptoms.

Table S4. Gender and age moderation of Hypothesis 1 and 2 associations.

| **Hypothesis** | **Outcome** | **Interaction term** | ***b*** | **SE** | ***p*** | **95% CI** | **N** |
| --- | --- | --- | --- | --- | --- | --- | --- |
| H1 | Traditional co-rumination | Problem-talk partners x gender | -0.03 | 0.03 | .302 | [-0.10, 0.03] | 538 |
| H1 | Traditional co-rumination | Problem-talk partners x age | -0.01 | 0.01 | .422 | [-0.03, 0.01] | 538 |
| H1 | Internalizing symptoms | Problem-talk partners x gender | -0.00 | 0.03 | .889 | [-0.06, 0.05] | 541 |
| H1 | Internalizing symptoms | Problem-talk partners x age | 0.00 | 0.01 | .974 | [-0.02, 0.02] | 541 |
| H1 | Friendship satisfaction/closeness | Problem-talk partners x gender | -0.07 | 0.04 | .098 | [-0.16, 0.01] | 534 |
| H1 | Friendship satisfaction/closeness | Problem-talk partners x age | 0.00 | 0.01 | .909 | [-0.03, 0.03] | 534 |
| H2 | Traditional co-rumination | Mean dyadic co-rumination x gender | -0.05 | 0.06 | .393 | [-0.17, 0.07] | 427 |
| H2 | Traditional co-rumination | Mean dyadic co-rumination x age | -0.00 | 0.02 | .978 | [-0.04, 0.04] | 427 |
| H2 | Internalizing symptoms | Mean dyadic co-rumination x gender | 0.00 | 0.07 | .946 | [-0.13, 0.14] | 428 |
| H2 | Internalizing symptoms | Mean dyadic co-rumination x age | -0.02 | 0.02 | .419 | [-0.06, 0.03] | 428 |
| H2 | Friendship satisfaction/closeness | Mean dyadic co-rumination x gender | -0.04 | 0.10 | .709 | [-0.23, 0.15] | 428 |
| H2 | Friendship satisfaction/closeness | Mean dyadic co-rumination x age | 0.01 | 0.03 | .855 | [-0.06, 0.07] | 428 |

*Note.* Gender and age moderation were tested simultaneously for each respective model. Each model included the main predictor (number of problem-talk partners for H1, mean dyadic co-rumination for H2), age, gender, and the two interaction terms (predictor X gender, predictor X age). These analyses were restricted to adolescents identifying as boys or girls, with boys as the reference group. All continuous variables were centered.

Table S5. Number of friends as an additional control variable for Hypothesis 1 associations.

| **Hypothesis** | **Outcome** | **Predictor** | ***b*** | **SE** | ***p*** | **95% CI** | **N** |
| --- | --- | --- | --- | --- | --- | --- | --- |
| H1 | Traditional co-rumination | Problem-talk partners | 0.05 | 0.02 | .005 | [0.02, 0.09] | 552 |
| H1 | Traditional co-rumination | Number of friends | 0.00 | 0.01 | .915 | [-0.02, 0.02] | 552 |
| H1 | Internalizing symptoms | Problem-talk partners | -0.04 | 0.02 | .022 | [-0.07, -0.01] | 557 |
| H1 | Internalizing symptoms | Number of friends | -0.02 | 0.01 | .042 | [-0.03, -0.00] | 557 |
| H1 | Friendship satisfaction/closeness | Problem-talk partners | 0.15 | 0.02 | < .001 | [0.10, 0.19] | 549 |
| H1 | Friendship satisfaction/closeness | Number of friends | -0.09 | 0.01 | < .001 | [-0.11, -0.07] | 549 |

*Note.* Number of friends was included as an additional control variable when testing Hypothesis 1 associations.

Table S6. CR2 robust standard error sensitivity analyses for Hypothesis 1 and 2.

| **Hypothesis** | **Outcome** | **Predictor** | **Model** | ***b*** | **95% CI** | ***p*** | **Consistent with main analysis** |
| --- | --- | --- | --- | --- | --- | --- | --- |
| H1 | Traditional co-rumination | Problem-talk partners | Base CR2 | 0.05 | [0.01, 0.09] | .011 | Yes |
| H1 | Traditional co-rumination | Problem-talk partners | Gender/age-adjusted CR2 | 0.05 | [0.02, 0.09] | .006 | Yes |
| H1 | Internalizing symptoms | Problem-talk partners | Base CR2 | -0.04 | [-0.07, -0.02] | .003 | Yes |
| H1 | Internalizing symptoms | Problem-talk partners | Gender/age-adjusted CR2 | -0.05 | [-0.07, -0.02] | .003 | Yes |
| H1 | Friendship satisfaction/closeness | Problem-talk partners | Base CR2 | 0.09 | [0.05, 0.14] | < .001 | Yes |
| H1 | Friendship satisfaction/closeness | Problem-talk partners | Gender/age-adjusted CR2 | 0.09 | [0.04, 0.14] | < .001 | Yes |
| H2 | Traditional co-rumination | Mean dyadic co-rumination | Base CR2 | 0.49 | [0.43, 0.55] | < .001 | Yes |
| H2 | Traditional co-rumination | Mean dyadic co-rumination | Gender/age-adjusted CR2 | 0.48 | [0.41, 0.54] | < .001 | Yes |
| H2 | Internalizing symptoms | Mean dyadic co-rumination | Base CR2 | 0.17 | [0.11, 0.23] | < .001 | Yes |
| H2 | Internalizing symptoms | Mean dyadic co-rumination | Gender/age-adjusted CR2 | 0.12 | [0.06, 0.17] | < .001 | Yes |
| H2 | Friendship satisfaction/closeness | Mean dyadic co-rumination | Base CR2 | 0.21 | [0.11, 0.30] | < .001 | Yes |
| H2 | Friendship satisfaction/closeness | Mean dyadic co-rumination | Gender/age-adjusted CR2 | 0.23 | [0.13, 0.32] | < .001 | Yes |

*Note.* CR2 models were estimated using linear models with school fixed effects and cluster-robust CR2 standard errors clustered by W1 classroom. The age/gender-adjusted models additionally included age and gender covariates. “Consistent with main analysis” indicates that association was consistent with the finding in the corresponding mixed model conducted for the main analyses.

Table S7. Classroom participation rate as a moderator of Hypothesis 1 and 2 associations.

| **Hypothesis** | **Outcome** | **Interaction term** | **Model** | ***b*** | **SE** | ***p*** | **95% CI** | **N** |
| --- | --- | --- | --- | --- | --- | --- | --- | --- |
| H1 | Traditional co-rumination | Problem-talk partners x classroom participation rate | Base model | -0.03 | 0.12 | .819 | [-0.25, 0.20] | 567 |
| H1 | Traditional co-rumination | Problem-talk partners x classroom participation rate | Gender/age-adjusted model | -0.05 | 0.11 | .674 | [-0.27, 0.17] | 552 |
| H1 | Internalizing symptoms | Problem-talk partners x classroom participation rate | Base model | 0.15 | 0.11 | .165 | [-0.06, 0.36] | 572 |
| H1 | Internalizing symptoms | Problem-talk partners x classroom participation rate | Gender/age-adjusted model | 0.15 | 0.10 | .117 | [-0.04, 0.35] | 557 |
| H1 | Friendship satisfaction/closeness | Problem-talk partners x classroom participation rate | Base model | -0.16 | 0.15 | .291 | [-0.45, 0.14] | 563 |
| H1 | Friendship satisfaction/closeness | Problem-talk partners x classroom participation rate | Gender/age-adjusted model | -0.15 | 0.15 | .332 | [-0.44, 0.15] | 549 |
| H2 | Traditional co-rumination | Mean dyadic co-rumination x classroom participation rate | Base model | -0.13 | 0.19 | .505 | [-0.50, 0.25] | 443 |
| H2 | Traditional co-rumination | Mean dyadic co-rumination x classroom participation rate | Gender/age-adjusted model | -0.15 | 0.19 | .438 | [-0.52, 0.23] | 436 |
| H2 | Internalizing symptoms | Mean dyadic co-rumination x classroom participation rate | Base model | -0.04 | 0.22 | .843 | [-0.48, 0.39] | 444 |
| H2 | Internalizing symptoms | Mean dyadic co-rumination x classroom participation rate | Gender/age-adjusted model | -0.06 | 0.21 | .760 | [-0.48, 0.35] | 437 |
| H2 | Friendship satisfaction/closeness | Mean dyadic co-rumination x classroom participation rate | Base model | -0.08 | 0.30 | .779 | [-0.68, 0.51] | 444 |
| H2 | Friendship satisfaction/closeness | Mean dyadic co-rumination x classroom participation rate | Gender/age-adjusted model | -0.10 | 0.30 | .731 | [-0.70, 0.49] | 437 |

*Note.* Classroom participation moderation was tested for each Hypothesis 1 and 2 association. Each model included the main predictor (number of problem-talk partners for H1, mean dyadic co-rumination for H2), classroom participation rate, and the interaction term (predictor X classroom participation rate). Gender/age-adjusted models additionally included gender and age covariates. Classroom participation rate was calculated as the number of participating adolescents in each classroom divided by classroom roster size. All continuous variables were centered.

Table S8. Simulation-based power for Hypothesis 1 and 2.

| **Hypothesis** | **Outcome** | **Predictor** | **N** | **β = .10** | **β = .15** | **β = .20** | **β = .25** | **β = .30** |
| --- | --- | --- | --- | --- | --- | --- | --- | --- |
| H1 | Traditional co-rumination | Problem-talk partners | 567 | 67.0% | 94.5% | 99.5% | 100.0% | 100.0% |
| H1 | Internalizing symptoms | Problem-talk partners | 572 | 69.5% | 97.0% | 99.5% | 100.0% | 100.0% |
| H1 | Friendship satisfaction/closeness | Problem-talk partners | 563 | 66.5% | 92.5% | 99.5% | 100.0% | 100.0% |
| H2 | Traditional co-rumination | Mean dyadic co-rumination | 443 | 80.5% | 97.5% | 100.0% | 100.0% | 100.0% |
| H2 | Internalizing symptoms | Mean dyadic co-rumination | 444 | 56.0% | 85.0% | 98.0% | 99.5% | 100.0% |
| H2 | Friendship satisfaction/closeness | Mean dyadic co-rumination | 444 | 61.0% | 92.0% | 99.5% | 100.0% | 100.0% |

*Note.* Power values are based on simulation-based sensitivity analyses using the same model-specific analytic samples, classroom clustering, and random-effects structure as the corresponding primary models. β values refer to standardized regression coefficients.
